# Real-time observation of conformational switching in single conjugated polymer chains

**DOI:** 10.1126/sciadv.aao5786

**Published:** 2018-02-16

**Authors:** Francisco Tenopala-Carmona, Stephanie Fronk, Guillermo C. Bazan, Ifor D. W. Samuel, J. Carlos Penedo

**Affiliations:** 1 Organic Semiconductor Centre, Scottish Universities Physics Alliance, School of Physics and Astronomy, University of St. Andrews, North Haugh, St. Andrews, Fife KY16 9SS, UK.; 2 Department of Materials and Chemistry and Biochemistry, University of California, Santa Barbara, Santa Barbara, CA 93106, USA.; 3 Biomedical Sciences Research Complex, University of St. Andrews, North Haugh, St. Andrews, Fife KY16 9SS, UK.

## Abstract

Conjugated polymers (CPs) are an important class of organic semiconductors that combine novel optoelectronic properties with simple processing from organic solvents. It is important to study CP conformation in solution to understand the physics of these materials and because it affects the properties of solution-processed films. Single-molecule techniques are unique in their ability to extract information on a chain-to-chain basis; however, in the context of CPs, technical challenges have limited their general application to host matrices or semiliquid environments that constrain the conformational dynamics of the polymer. We introduce a conceptually different methodology that enables measurements in organic solvents using the single-end anchoring of polymer chains to avoid diffusion while preserving polymer flexibility. We explore the effect of organic solvents and show that, in addition to chain-to-chain conformational heterogeneity, collapsed and extended polymer segments can coexist within the same chain. The technique enables real-time solvent-exchange measurements, which show that anchored CP chains respond to sudden changes in solvent conditions on a subsecond time scale. Our results give an unprecedented glimpse into the mechanism of solvent-induced reorganization of CPs and can be expected to lead to a new range of techniques to investigate and conformationally manipulate CPs.

## Teaser

Single-molecule experiments in organic solvents provide new insight into the conformational dynamics of conjugated polymers.

## INTRODUCTION

Conjugated polymers (CPs) are macromolecular structures used in different types of optoelectronic devices including organic light-emitting diodes, organic transistors, and solar cells (*1*, *2*). Despite their growing potential in modern optoelectronics, a fundamental understanding of how the conformation of a polymer chain in solution affects its electronic and photophysical properties still constitutes an important and challenging problem in polymer nanoscience and soft-matter optoelectronic engineering (*3*–*6*). Because conformations can vary from chain to chain, the observation of single polymer chains embedded in host matrices, such as polystyrene (PS) and poly(methyl methacrylate) (PMMA), has emerged as a powerful method to study the relationship between polymer shape and optoelectronic properties without interference from ensemble-averaging (*7*–*10*). Unfortunately, these techniques restrict the conformational flexibility of the polymer chains and do not allow real-time variations in organic solvent environments (*11*, *12*).

The closest steps toward measuring the photophysics of single chains that undergo controlled conformational changes have been achieved by momentarily releasing the conformational constraints imposed by the host matrices via solvent vapor annealing (SVA) (*13*) or by decreasing the diffusion speed of CP chains in solvent-based environments by suspending them in liquid crystals (*14*) or viscous solutions (*15*, *16*). However, these approaches are limited either by the time that is necessary for SVA to occur, or by the trade-off between mimicking a good-solvent environment while still being able to track diffusing chains. As a result, the shape and conformational dynamics of individual CPs in organic solvent environments remain unknown. Instead of a conceptual microscopy limitation, the lack of methods to investigate single CPs in solution arises mostly from solvent-related technical challenges (*11*). These include the need for surface-anchoring methods that can resist prolonged exposure to organic solvents and the design of oxygen-free sample chambers that allow active flow manipulation while being sufficiently robust to solvent corrosion (*11*, *12*).

In response to the challenges described above, we introduce here a single-molecule microscopy method that allows, for the first time, to measure the photophysics of single surface-anchored CP chains adopting different conformations in 100% organic solvent environments. Using poly(3-hexylthiophene) (P3HT) as the model polymer (*17*, *18*), we further show, for the first time, how CP chains reorganize their conformation within seconds in response to sudden changes in solvent quality. This dynamic information is currently inaccessible by other methods. We demonstrate that when CPs are anchored at one end, and free to explore their structural landscape in the rest of the chain, they exhibit not only a high level of conformational heterogeneity from polymer to polymer but also within individual chains.

We first describe methods to synthesize triethoxysilane-terminated polymer chains that can be covalently anchored at one end to the glass surface, and thus exposed to any solvent condition, and the development of oxygen-free sample chambers compatible with organic solvents. We also show that our technique allows for measuring the photophysics of single chains as a function of solvent conditions without the interference from aggregation effects, even in solvents where the polymers chains are virtually insoluble—a feature that cannot be easily attained by any other technique.

The strategy described here provides a new experimental framework for studying the relationship between the conformation and the photophysics of CPs, as well as the nature of dynamic processes in these materials. We expect this technique to provide more detailed information about polymer morphology and dynamics in a wide set of solvent environments, improving our understanding of these materials.

## RESULTS AND DISCUSSION

### Design and synthesis of triethoxysilane-terminated P3HT chains

P3HT is considered a model system for CPs and is therefore a good initial stage for single-molecule, solution-based studies (*17*–*20*). The first step to develop an organic solvent–based approach and observe single P3HT chains for long periods of time requires anchoring individual chains at one end to avoid rapid diffusion while preserving their ability to respond to changes in solvent conditions. In an aqueous solution, this can be easily achieved using a protein-based approach that takes advantage of the specific noncovalent interaction between surface-anchored streptavidin proteins and biotin groups placed at the observed molecule (*12*, *21*, *22*). For instance, Dalgarno *et al.* (*12*) demonstrated the feasibility of this approach to monitor the fluorescence emission from biotin-derivatized poly(3-dodecylthiophene) chains in water. However, this strategy is incompatible with organic solvents that denature the protein, and therefore, we explored alternative methods based on the direct covalent attachment of the polymer to the glass surface. A triethoxysilane group was chosen on the basis of its well-known ability to covalently react with quartz and glass substrates (*23*, *24*). The incorporation of this functional group at one end of the chain provides CPs with the ability to be directly anchored to the substrate surface while preserving the necessary conformational freedom in the rest of the chain to respond to changes in solvent conditions.

**Fig. 1:**
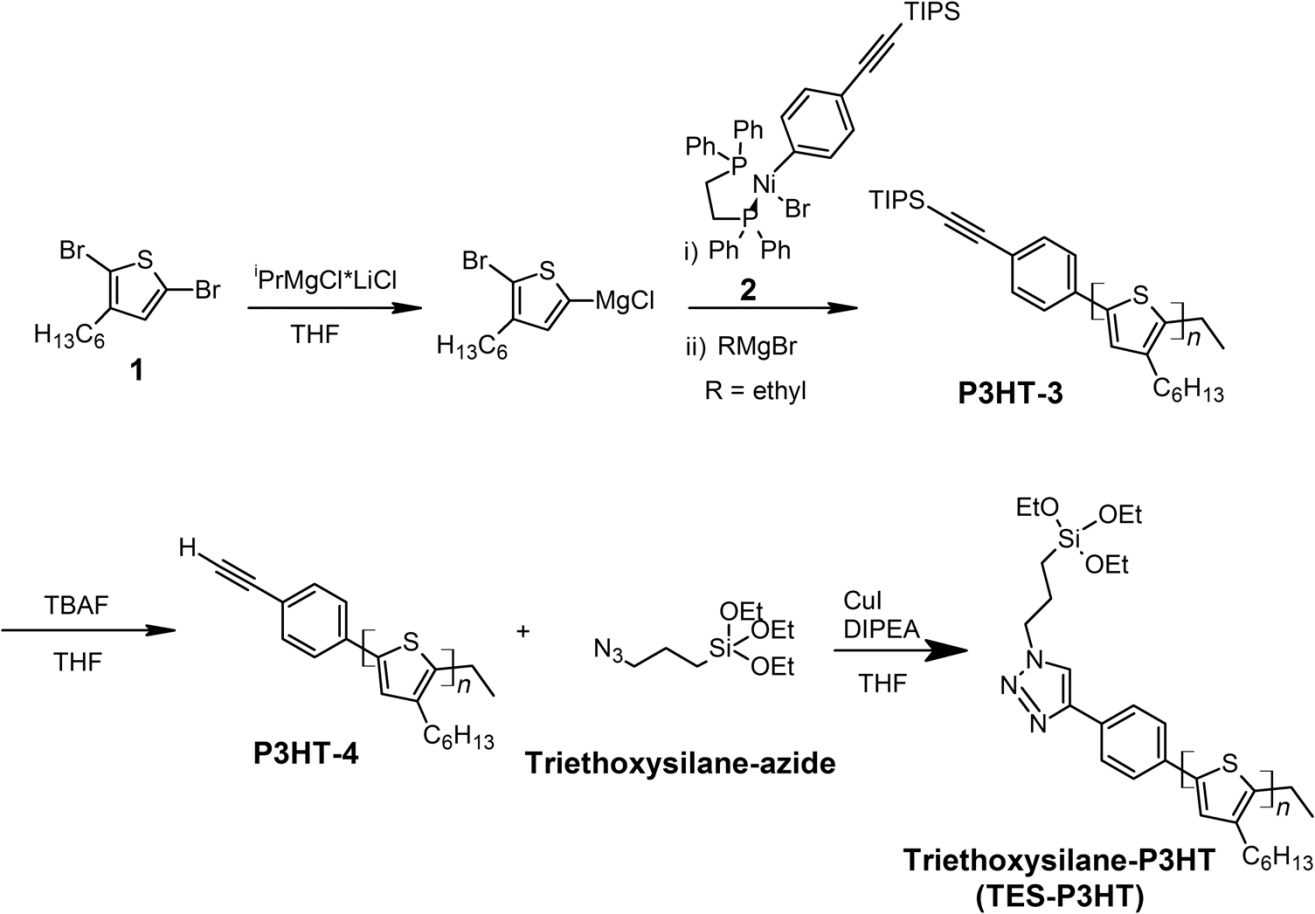
Synthesis of triethoxysilane-terminated P3HT (TES-P3HT) chains.

Specifically, P3HT obtained via Kumada catalyst-transfer polycondensation methods exhibits a well-defined, regioregular character with low dispersity (*25*, *26*). The molecular weights obtained using this method can be modulated by tuning the monomer-to-catalyst ratio, and the selection of the nickel catalyst determines the identity of the end group incorporated onto a single chain end (*27*). Initiator **2** was synthesized in two steps from (dppe)NiCl_2_ (Fig. 1) (*12*, *28*). A relevant feature of **2** is that it contains a triisopropylsilane (TIPS)–protected alkyne that allows post-polymerization functionalization via click chemistry. Controlled polymerization was carried out as shown in Fig. 1. We synthesized 2,5-dibromo-3-hexylthiophene according to literature methods (*29*), and we then treated it in tetrahydrofuran (THF) with 0.95 equivalent (eq.) of isopropylmagnesium chloride–lithium chloride complex to produce the reactive monomer. This solution was quickly added to a stirring solution of **2** at specific monomer-to-catalyst ratios. The polymerization was quenched with ethylmagnesium bromide to provide **P3HT-3** with a number-average molecular weight, *M*_n_, of 7.1 and 11.8 kDa and a dispersity, *Đ*, of 1.3 and 1.4 for 40:1 and 80:1 monomer-to-catalyst ratios, respectively. The identity of the end groups was confirmed by ^1^H nuclear magnetic resonance (NMR) spectroscopy and matrix-assisted laser desorption/ionization–time-of-flight (MALDI-TOF) mass spectrometry using an analysis method described previously (see the Supplementary Materials) (*30*, *31*).

The TIPS-protecting group was removed under basic conditions using tetrabutylammonium fluoride (TBAF) in THF at room temperature to yield **P3HT-4** for both chain lengths. ^1^H NMR spectroscopy confirms the removal of the TIPS-protecting group through the disappearance of the peak at 1.1 parts per million (ppm) and the appearance of the ethynyl peak at 3.1 ppm (fig. S2). **P3HT-4** was reacted with excess triethoxysilane-azide under click conditions comprising CuI and *N*,*N*-diisopropylethylamine (DIPEA) with THF as the solvent (*32*, *33*). The triethoxysilane-group was successfully incorporated onto one end of the polymer chain to produce triethoxysilane-P3HT (**TES-P3HT**), as confirmed by ^1^H NMR spectroscopy (section S1) and MALDI-TOF (fig. S3). Two polymers with different molecular weights were synthesized using this approach, **TES-P3HT-S** (*M*_n_ = 8770 Da, *M*_w_ = 9860, *Đ* = 1.1) and **TES-P3HT-L** (*M*_n_ = 10,600, *M*_w_ = 15,900, *Đ* = 1.5). The presence of triethoxysilane at a single chain end activates the polymer for surface attachment to a quartz or glass substrate.

### Surface-anchoring of CP chains in organic solvents

The use of single-molecule fluorescence techniques in organic solvents still lags behind its application for biological systems, where for more than a decade, it has been rewriting our understanding of biomolecules. This is mostly due to technical challenges derived from the intrinsic properties of organic solvents compared to water (*11*). For instance, sample chambers for single-molecule studies are commonly sealed with double-sided tape (*34*) or nail polish (*35*) that dissolves in organic solvents. In addition, aspects such as solvent impurities and oxygen removal, where a range of protein-based oxygen scavengers can be used, are easier to deal with in aqueous solution (*36*, *37*). Significant progress has been made during the last 10 years toward oxygen-tight and solvent-resistant chambers for single-molecule microscopy. For example, Ameloot *et al.* (*38*) developed an oxygen-tight brass chamber for studying catalytic cycles in acidic and basic aqueous conditions, Upadhyay *et al.* (*24*) developed an all-glass chamber for studying dendrimeric molecular catalysts in organic solvents, and Feng *et al.* (*39*) developed a reaction chamber that allowed single-molecule experiments in THF. However, the chambers developed by Ameloot *et al.* (*38*) and by Feng *et al.* (*39*) were sealed with rubber and Devcon 5-min epoxy, respectively, which do not have excellent resistance to a wide range of organic solvents. In this regard, an all-glass chamber similar to the one developed by Upadhyay *et al.* (*24*) presents a good alternative; however, it is not reusable and is time-consuming to build. Thus, a chamber that allows for the active control of solvent flow with excellent resistance to a wide range of organic solvents and is reusable and easy to mount still needs to be developed.

**Fig. 2:**
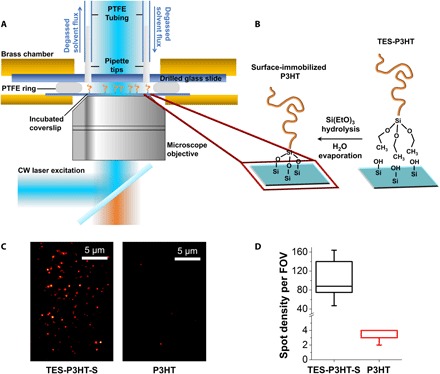
Monitoring single P3HT chains in organic solvents. (**A**) Diagram of the sample chamber designed for single-molecule imaging in organic solvents including details of the injection system and the epifluorescence excitation and emission detection pathways. Components are not drawn to scale. (**B**) Schematic of the surface-anchoring strategy to covalently anchor single-P3HT chains to the glass substrate. (**C**) Representative single-molecule images of triethoxysilane-modified P3HT (left) and unmodified P3HT (right). (**D**) Box-plot comparison of the density of fluorescent spots observed using TES-P3HT (black) and unmodified P3HT (red). The extremes, upper and lower quartiles of the distribution, and the median are represented by the whiskers, box and middle lines, respectively. The whisker and upper quartile relative to sample P3HT are at the same value as the median. A 20-fold higher density of spots obtained for TES-P3HT compared to P3HT confirms specific covalent attachment of single chains via the silane end group. FOV, field of view.

To address these challenges, we developed a solvent-resistant and oxygen-free chamber that allows real-time solvent exchange through two injection holes drilled on the microscope slide. The chamber, schematically shown in Fig. 2A, was made by creating a gap between a fused-silica slide and a polymer-incubated coverslip via a polytetrafluoroethylene (PTFE) ring. This chamber has the advantage of being sealed only by applying pressure via two brass plates, and it was tested to be robust against leakage and solvent evaporation. All experiments reported in this work were carried out using this sample chamber. Notably, the design of this chamber is similar to the one developed by Ameloot *et al*. (*38*). However, our ring is made of PTFE, which is resistant to most organic solvents used in the fabrication of conjugated polymer–based devices (*40*) and, like rubber, is commercially available.

Covalent attachment of silane derivatives to glass surfaces, particularly the silane-terminated polyethylene glycol, is a common method to passivate glass surfaces, and it is fully compatible with the use of organic solvents (*41*). Furthermore, its use in surface-anchoring organic small molecules and catalysts has been proven in organic solvent environments (*38*). Here, we optimized these surface-functionalization protocols to account for polymer solubility. Complete details of the method are described in Materials and Methods. In brief, an initial hydrolysis step under acidic conditions was applied to the TES-P3HT chains for removing the terminal ethoxy of the TES group. This reaction was followed by contact with plasma-etched substrates. Plasma etching was used to activate the glass surface with sufficient hydroxyl groups that can efficiently react with triethoxysilane functional groups. Finally, the residual solvent was evaporated in vacuum. This reaction is schematically summarized in Fig. 2B. To ensure that nonspecifically bound (adsorbed) chains are completely removed, we rinsed the P3HT functionalized glass substrates thoroughly in *o*-dichlorobenzene (*o*-DCB) for 1 min in an ultrasound bath.

The efficiency and specificity of the surface-anchoring protocol was tested by comparing two P3HT samples of the same molecular weight but with only one of them carrying the reactive triethoxysilane group. Both samples were incubated under the same experimental conditions and were imaged under continuous illumination using a custom-built wide-field single-molecule microscope operating in epifluorescence mode and using an electron-multiplying charge-coupled device camera for fluorescence detection (see Materials and Methods). By using this microscope configuration, it is possible to monitor several individual chains simultaneously during extended periods of time (minutes), provided that they are spatially separated. For the triethoxysilane-terminated polymer (**TES-P3HT-S**), the single-molecule images in *o*-DCB showed fluorescent spots that remain static during the whole measurement (Fig. 2C, left). By contrast, incubation of the glass surface with **P3HT** polymers lacking end-functionalization showed a much lower number of fluorescence spots per field of view (~70 μm × 115 μm) (Fig. 2C, right). We estimated an ~20-fold higher density of fluorescent spots within an identical size field of view when using **TES-P3HT-S** instead of **P3HT** (Fig. 2D), thus confirming the specific anchoring of **TES-P3HT-S** polymer chains on the glass surface via the terminal triethoxysilane group.

Note that CPs exhibit a high propensity to form aggregates in poor-quality solvents (*42*). Because of this propensity to aggregate, unambiguously assigning every fluorescent signal as arising from a single polymer instead of a single aggregate has been far from trivial in single-molecule experiments (*43*, *44*). Our strategy allowed us to rule out the presence of these aggregates due to the specific-binding approach. Once the anchoring reaction is completed, the samples can be imaged in any solvent of choice without the risk of multichain aggregation. Thus, the surface-anchoring approach also enabled us to visualize, for the first time, single chains in solvents where the polymer has extremely poor solubility, thus giving access to self-aggregation studies of single chains that were previously unavailable.

### Single-molecule classification of P3HT photoluminescence profiles in organic solvents

In an initial experiment, single triethoxysilane-terminated **TES-P3HT-L** polymers were anchored on a glass coverslip and separately imaged in two different solvents, namely, *o*-DCB and dimethyl sulfoxide (DMSO). These solvents are known for being a “good” and a “poor” solvent for P3HT, respectively (*45*, *46*). Here, we have adopted the same terminology as commonly found in the CP literature, where the good solvent terms refers to those where the polymer material is highly soluble and polymer chains adopt mostly an extended conformation. On the other hand, very limited solubility and collapsed chains are characteristics of poor solvents (*47*, *48*). *o*-DCB and DMSO have solubility values for P3HT of ~14.7 mg/ml and <0.1 mg/ml, respectively (*46*, *49*); therefore, they constitute excellent examples of good- and poor-quality solvents. Surface-anchored **TES-P3HT-L** chains were excited with a 473-nm laser operating in continuous wave mode and imaged using wide-field epifluorescence. A wavelength of 473 nm has been shown to correspond to an isosbestic point in mixtures of good and poor solvents and thus ensures identical absorption efficiency in the solvents investigated (*50*). To enhance the signal-to-noise ratio of the intensity measurement and to decrease the photobleaching rates, all the experiments were carried out in degassed, spectroscopy-grade solvents.

**Fig. 3:**
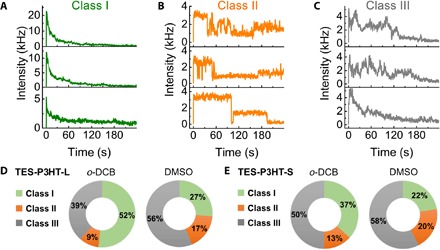
Single-molecule analysis of surface-anchored TES-P3HT-L polymers in good (*o*-DCB) and poor solvents (DMSO). Single-molecule PL intensity traces were classified into three classes (I to III). (**A**) Representative single-molecule class I traces obtained for TES-P3HT-L displaying a continuous decrease in PL intensity. (**B**) Representative single-molecule class II traces. Class II traces were characterized by multilevel fluctuations in PL intensity. (**C**) Representative single-molecule class III traces obtained for TES-P3HT-L displaying a hybrid PL profile combining regions of continuous decrease in PL intensity and regions with multilevel fluctuations. Statistical contributions of each class obtained in *o*-DCB (good solvent) and DMSO (poor solvent) are shown for TES-P3HT-L (**D**) and TES-P3HT-S (**E**).

Examples of single-molecule photoluminescence (PL) intensity traces collected in both solvents are shown in Fig. 3. Three main categories (classes I to III) were identified according to their time-dependent intensity profiles (Fig. 3). Class I comprises traces characterized by an initial high-intensity level that monotonically decays either to noise level (Fig. 3A, top and middle) or to a low-intensity stable value above noise (Fig. 3A, bottom). Most of these traces exhibited an exponential-like decay with a half-life of ~30 s, and in some of them, a stepwise decrease in intensity was observed (fig. S8).

The second category (class II) refers to traces exhibiting multilevel intensity fluctuations (blinking) between clear levels (Fig. 3B). The average initial intensity of the traces included in this category was between three and four times lower than those included in class I (fig. S9). Because the excitation wavelength corresponds to an isosbestic point (*50*), implying no variation in the absorption cross section between both solvents, this lower intensity is exclusively due to a decrease in the PL quantum yield (PLQY) of single chains. A similar decrease in intensity was reported for P3HT chains embedded in a PMMA matrix compared to Zeonex (*48*). Another clear difference of class II traces compared to class I was the heterogeneity between different intensity profiles. For instance, the frequency and dwell time of these “blinking” events varied greatly from trace to trace, and complete photobleaching was only observed for some traces within the 240-s acquisition window (Fig. 3B).

Last, those PL intensity traces showing a combination of features, resembling those characteristics of classes I and II categories were grouped separately (class III). Representative traces within this group are shown in Fig. 3C. These combinations presented an initial rise followed by fast decays in the PL intensity or multilevel fluctuations with no clear intensity levels until complete chromophore photobleaching occurred (Fig. 3C). Examples of each category were found in both *o*-DCB and DMSO, although their relative contribution differs between them (Fig. 3D). In *o*-DCB, ~52% of the **TES-P3HT-L** traces exhibited a fast intensity decay (class I), whereas only 39% exhibited a hybrid PL profile (class III) (Fig. 3D). When DMSO was used as solvent, the proportion of class I traces decreased to 27% and most traces showed a class III hybrid (~56%).

### Conformational heterogeneity of surface-anchored P3HT chains in organic solvents

Blinking and photobleaching are commonly observed phenomena in single-molecule microscopy studies of CPs. These two phenomena have been widely studied and related to the energy-transfer properties of single CP chains. From our results, the higher contribution of class I profiles in degassed *o*-DCB (~52%) compared to DMSO (~27%) suggests a direct correlation between efficient solvation of the polymer chains in a good solvent and fast PL intensity decay. This correlation has been previously proposed for P3HT and poly(2-methoxy-5-(2-ethylhexyloxy)-1,4-phenylenevinylene (MEH-PPV) polymers embedded in a host matrix (*9*, *10*, *48*, *51*). In the widely accepted picture, fast exponential-like photobleaching (class I) is associated with disordered chains with a large number of conjugated segments across which energy transfer does not occur easily. This poor communication between chromophores leads to stochastic photobleaching of the segments, which is reflected in the exponentially decaying profile. In a previous work by Onda *et al.* (*15*), this exponentially decaying profile was also observed in single MEH-PPV chains adsorbed on glass substrates while in contact with a 20:80 (m/m) PS/toluene solution. By contrast, chains that are freely diffusing in the same solution exhibited a fivefold decrease in the photobleaching rates. Thus, Onda *et al.* concluded that fast bleaching was due to conformational constraints imposed by adsorption. Here, we cannot discard interactions with the substrate due to the surface-anchoring approach. However, clear changes in the behavior of chains going from *o*-DCB to DMSO suggest that any possible constraint other than the tethered end of the chains is negligible. This is further supported by the absence of anchored chains when the triethoxysilane agent was not incorporated (Fig. 2, C and D). The fact that 27% of the chains in DMSO exhibited class I behavior implies that this can also be associated with collapsed conformations. Thus, we associate class I traces to disordered configurations, either extended in *o*-DCB or collapsed in DMSO. Examples of these conformations can be the so-called random coil and molten globule, respectively (*52*).

Traces exhibiting multilevel blinking between clearly defined levels (class II) have been commonly assigned to highly folded and ordered configurations adopted by CP chains in inert matrices mimicking poor solvents (*48*, *53*). In those conformations, efficient energy transfer leads to emission from a low number of chromophores that are continuously fed by their neighbors (*9*, *10*). Thus, the efficient funneling of excitons reduces the photobleaching rates and the number of PL intensity levels. In our experiments, this kind of traces account for less than 17% of the total in either solvent (Fig. 3D). We observed a clear switching from class I as the predominant intensity profile in *o*-DCB (52%) to class III in DMSO (~56%) (see Fig. 3D, right).

This trend can be explained by an intermediate degree of disorder in the conformation of single chains. The random, fast fluctuations observed in class III traces suggest a large number of chromophores undergoing reversible transit to dark states. This behavior has been previously observed and associated with localized charges in single P3HT chains (deep exciton traps) (*54*). By contrast, the blinking from more ordered chains exhibiting class II behavior is more likely to be caused by quenching from delocalized charge states (shallow exciton traps). These dark states have been associated with photogenerated charges (*7*, *47*) or with the interaction of the polymer chains with quenchers localized on the surface of the substrate (*15*, *38*).

Because of the restricted conformational freedom that single chains are expected to have in poor solvents, we associate the class III behavior in DMSO to collapsed conformations with sufficient disorder to present mainly localized charge states but insufficient to undergo fast photobleaching as class I traces. However, it is interesting to note that the time scale for the fluctuations observed in the class III traces (approximately tens of seconds) is close to the temporal variation detected in the Raman spectra of single MEH-PPV chains due to backbone twisting (*55*). Blinking in this time scale was also associated with the dynamics in MEH-PPV chains in PS/toluene solutions in the aforementioned work by Onda *et al.* (*15*). Thus, the degree of conformational freedom allowed by single-end anchoring in organic solvents can enable this kind of dynamics not only in a good solvent like *o*-DCB but also probably in DMSO.

Our current data do not allow us to firmly confirm whether class III traces represent P3HT chains dynamically exchanging between different conformational states or localized quenchers in disorderly collapsed chains. However, discriminating between both scenarios should be possible by adapting our technique for spectral and polarization modulation depth measurements.

### Influence of polymer length on the PL properties of single P3HT chains in organic solvents

Intuitively, the degree of intrachain heterogeneity resulting from different conformational domains coexisting within single chains could be influenced by polymer length (*56*, *57*). To explore this aspect, we synthesized a triethoxysilane-terminated P3HT sample with a shorter length (**TES-P3HT-S**) (*M*_n_ = 8770 Da, *M*_w_ = 9860, *Đ* = 1.1). We observed a significant decrease (~50%) in the average initial PL intensity of **TES-P3HT-S** chains compared to **TES-P3HT-L** in *o*-DCB, and this effect was more pronounced for class I PL profiles followed by classes III and II (fig. S9). This difference between both polymers can be rationalized by simply considering that **TES-P3HT-L** may contain a higher number of simultaneously emissive chromophores compared to **TES-P3HT-S** that leads to a higher initial PL intensity. In addition, switching from classes I to III categories between *o*-DCB (Fig. 3E, left) and DMSO (Fig. 3E, right) was less pronounced for **TES-P3HT-S** than for **TES-P3HT-L**. For the shorter polymer, class III was the predominant profile in both solvents (50 to 60%), and only a slight decrease from 37 to 22% was observed for class I when changing from *o*-DCB to DMSO (Fig. 3E). The distribution of categories in DMSO was almost identical between **TES-P3HT-L** and **TES-P3HT-S** (Fig. 3, D and E, right), suggesting that a similar distribution of conformations was adopted by both polymers in a poor-solvent environment. This can be rationalized if we consider that the persistence length of poly(3-alkylthiophenes) has been shown to range from ~2 to 3 nm in *o*-DCB to ~1 to 1.5 nm in toluene, a solvent with lower solubility (~0.7 mg/ml) (*58*). Thus, the low–persistence length values suggest that any potential length effect might be overridden by the intrinsic flexibility of the chain when it is forced to collapse by its interaction with a poor solvent. Moreover, the low contribution of class II category observed in both solvents and for both polymer lengths investigated agrees with the interpretation of this material as a very flexible polymer that very rarely adopts exclusively rod-like structures.

Finally, it is important to note that there was a low but nonzero percentage of traces exhibiting class II behavior in *o*-DCB, which would not be expected when the chain is allowed to interact with a good solvent. We reason that this is due to chains containing a very small number of chromophores. Although most of the single chains we study can contain multiple chromophores, the shorter chains may have conjugation lengths that allow only one or two chromophores. This smaller number of chromophores in shorter chains can explain the higher fraction of classes II and III traces for sample **TES-P3HT-S** in *o*-DCB when compared to the same experiment for **TES-P3HT-L**. Conjugation lengths of dimensions similar to those of the whole chains that contain them have been estimated in previous bulk-dilution studies of P3HT (*50*). Furthermore, it has been previously shown that conjugation in single chains may not be broken even in partially bent conformations (*59*), which would be in agreement with this interpretation.

### Real-time conformational switching of single-end–anchored P3HT chains

Single-end anchoring of single chains enables a new type of experiment in which different conformations of single chains can be actively induced while simultaneously measuring its PL output, simply by performing an exchange of solvents in real time. Because the polymer is anchored on the glass surface only at one end, it can reorganize its conformation in response to abrupt changes in solvation conditions. This rapid solvent-exchange experiment can enable new understanding of polymer conformational dynamics that is inaccessible through traditional ensemble-averaging techniques and through current single-molecule approaches using SVA after matrix-based immobilization.

**Fig. 4:**
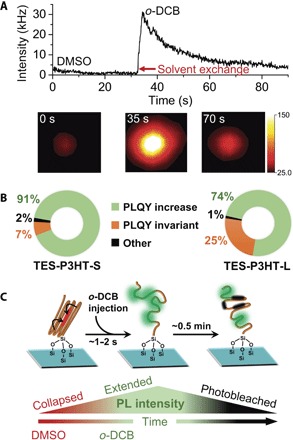
Conformational switching of single CP chains using real-time solvent-switching methods. (**A**) Representative single-molecule PL intensity trace showing the abrupt change in fluorescent emission taking place in the same TES-P3HT-S chain upon switching at ~31 s from DMSO to *o*-DCB. A >5-fold change in fluorescent emission is followed by a continuous monotonic decrease in signal due to chromophore photobleaching. Images in the lower panel show two-dimensional (2D) intensity distributions of emitted light from the same P3HT chain as shown in the upper panel taken at *t* = 0 s in DMSO (left), *t* = 35 s in *o*-DCB (middle), and at *t* = 70 s in *o*-DCB (right). (**B**) Percentage distribution of single chains that displayed an increase (green), decrease (orange), or no variation (black) in intensity during solvent-switching for TES-P3HT-S (left) and TES-P3HT-L (right) polymers. (**C**) Schematics of the collapsed-to-expanded transition observed during the solvent-exchange experiment from a poor solvent (DMSO) to a good solvent (*o*-DCB) shown in (A). The bottom panel summarizes the interplay between PL intensity, solvent-induced change in conformation and photobleaching processes as a function of time.

To prove the feasibility and potential of this solvent-induced conformational switching, we designed an experiment where surface-anchored **TES-P3HT-S** chains were firstly imaged in a poor solvent (DMSO) for a time period of ~30 s before switching to a good solvent environment (*o*-DCB), while collecting PL intensity data. On the basis of our previous observations (Fig. 3), where continuous excitation in a good solvent predominantly induces fast photobleaching of individual chromophores within the chain, we decided to initially induce a poor-to-good solvent transition to avoid excessive photobleaching before solvent switching. A representative example of the PL profile obtained when switching from DMSO to *o*-DCB is shown in Fig. 4A (the corresponding movie S2 is available in the Supplementary Materials). Injection of *o*-DCB, replacing DMSO, induced an initial sudden increase in the PL intensity (~5- to 10-fold) of the anchored **TES-P3HT-S** chain, followed by an exponential-like decay with a half-life of ~36 s. Similar PL intensity profiles were obtained for single **TES**-**P3HT-L** chains (fig. S10 and movie S1), but the percentage of polymer chains exhibiting a sudden PL increase upon solvent exchange was slightly lower (~71%) than for **TES-P3HT-S** (~91%) (Fig. 4B). Considering the previous single-solvent experiments where P3HT polymers were imaged in *o*-DCB and DMSO separately (Fig. 3), we interpreted the sudden increase in PLQY as evidence for DMSO being replaced by *o*-DCB with the subsequent transition from a collapsed to stretched conformation.

Only ~2% of **TES-P3HT-S** and ~1% of **TES**-**P3HT-L** chains exhibited either a slight increase or even a quenching of its PL intensity upon solvent exchange (Fig. 4B). The fact that the longer polymer, **TES-P3HT-L**, showed a more heterogeneous behavior with a higher proportion of chains being unaffected by solvent exchange suggests that the immediate response of the polymer chain to the environment is not only controlled by the chemical composition of the polymer and the nature of the solvent but also, to a certain degree, by polymer length. Likewise, chains exhibiting only a slight increase in the PL intensity may have had conformational stability along only certain folded regions, whereas another short, free region was responsible for the small spike in the intensity traces. The few chains that exhibited quenching may have had significant portions already photobleached during injection, which may explain the sudden decrease in intensity.

**Fig. 5:**
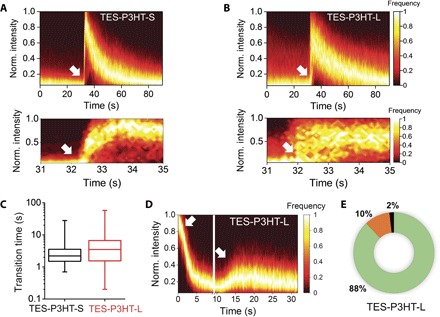
Single-molecule analysis of real-time solvent switching events as a function of P3HT polymer length. (**A**) Contour plot generated for TES-P3HT-S polymers by overlapping of 312 single-molecule PL intensity traces obtained during DMSO–to–*o*-DCB solvent-switching experiments. *o*-DCB was injected at 32 s (white arrow). Bottom: Detail of the progressive increase in PL intensity during the first seconds after solvent switching. (**B**) Contour plot generated for TES-P3HT-L polymers by overlapping of 335 single-molecule PL intensity traces obtained during DMSO–to–*o*-DCB solvent-switching experiments. *o*-DCB was injected at 32 s (white arrow). Bottom: Detail of the progressive increase in PL intensity during the first seconds after solvent switching. (**C**) Box plot showing the distribution of transition times from low-PL (DMSO) to high-PL (*o*-DCB) intensity values obtained for TES-P3HT-S (black) and TES-P3HT-L (red). (**D**) Contour plot generated for TES-P3HT-L polymers by overlapping 225 single-molecule traces obtained during two consecutive solvent-switching steps (white arrows) from *o*-DCB to DMSO and back to *o*-DCB. (**E**) Percentage distribution of single TES-P3HT-L chains that displayed an increase (green), decrease (orange), or no variation (black) in PLQY during the DMSO–to–*o*-DCB solvent-switching step shown in (D). Norm., normalized.

We reasoned that in longer chains, polymer segments can organize themselves into structures with higher packing and more stable configurations that are more difficult to disentangle by solvent exchange. Additional experimental evidence for a higher level of dynamic heterogeneity in **TES-PH3T-L** compared to **TES-P3HT-S** was obtained by superimposing all PL intensity traces for each of them, using the sharp rise in emission intensity as reference time for synchronization (Fig. 5, A and B, top). The contour plots generated for **TES-P3HT-S** (Fig. 5A) and **TES-P3HT-L** (Fig. 5B) during the real-time solvent switching confirmed substantial differences in the dynamic response of both polymers. For the shorter **TES-P3HT-S** polymer, the rise phase was relatively homogeneous (Fig. 5A, bottom), with an average switching time from the low to the maximum PLQY value of ~2.7 s, and most switching times were lower than 6 s (Fig. 5C). By contrast, **TES-P3HT-L** chains displayed a higher heterogeneity in the times that were needed for reaching the peak in PL intensity (Fig. 5B, bottom). We obtained an average **TES-P3HT-L** switching time of ~5 s (Fig. 5C), but now, the distribution of values was significantly broader and ranged from 1 to ~17 s (Fig. 5C). In both polymers, the average switching interval is higher than the time required for injection, which we estimated to be less than 1 s. Intuitively, a higher dynamic disorder for **TES-P3HT-L** compared to **TES-P4HT-S** could be expected because its higher contour length makes it more likely to have ordered, folded regions and agrees with the higher heterogeneity observed for the former in both *o*-DCB and DMSO (Fig. 3E).

To further explore the response of surface-anchored P3HT chains to sudden changes in solvation, we next performed two-step solvent-switching experiments. In these experiments, an initial solvent environment was replaced and then restored following two sequential real-time injections. A representative example using **TES-P3HT-L**, where *o*-DCB was replaced by DMSO and switched back to *o*-DCB, is shown in Fig. 5D. In these experiments, only a partial recovery of the initial PL intensity was observed because of the significant photobleaching of the anchored chain during the initial *o*-DCB imaging step. However, it is still possible to conclude from these data that the switching between polymer conformations is a reversible process. During the second injection step switching from DMSO to *o*-DCB, we observed a slight increase in the number of **TES-P3HT-L** chains responding to solvent exchange (~88%) (Fig. 5E) compared to ~71% observed in the single-injection experiment (Fig. 4B, right box plot). At this stage, we cannot completely rule out that a small drag force component, intrinsic to the injection event, might contribute to stretch the polymer and help to unfold trapped structures during the second switching step. However, even if present, this force component must be small, because it cannot prevent most of the chains from collapsing when switching from an *o*-DCB to a DMSO environment (fig. S11). The reversible equilibration of the polymer shape in response to changes in the surrounding environment is consistent with the exchange folding/unfolding events observed for MEH-PPV polymers during SVA (*13*). Moreover, no significant correlation in peak intensity or switching time was observed during the *o*-DCB/DMSO/*o*-DCB switching sequence, suggesting that as observed in single-molecule SVA, good solvents “reset” the polymer structure and cause the chain to lose memory of its initial conformation.

## CONCLUSION

We have successfully demonstrated single-molecule fluorescence microscopy of surface-anchored CPs in organic solvents. By using triethoxysilane functionalization at one end of the polymer, we ensured a specific anchoring of single chains to the glass surface while maintaining their ability to adapt to changes in solvent environment. The application of this method to the specific case of P3HT revealed a significant degree of inter- and intrachain conformational heterogeneity, as well as how it depends on solvent quality and polymer length. The development of specific single-chain anchoring methods compatible with organic solvents allowed us to study, for the first time, single P3HT chains adjusting their conformation in real time on a subsecond time scale in response to sudden changes in the solvation conditions. The measurements provide an exciting new way to probe polymer-solvent interactions and the self-aggregation mechanism of CPs in real time. Furthermore, our approach provides a pathway to combining fluorescence spectroscopy and direct mechanical manipulation of single CP chains in organic solvents.

## MATERIALS AND METHODS

### Reagents and materials for the synthesis of triethoxysilane-terminated P3HT

All reagents and solvents were purchased from Fisher Scientific, Sigma-Aldrich, Acros Organics, or BDM and used without further purification unless specified. Triethoxysilane-azide was purchased and used without further purification from Oakwood Chemicals. We purchased 1,2-bis(diphenylphosphino)ethane nickel (II) chloride from Strem Chemicals and used it as received. We synthesized 2,5-dibromo-3-hexylthiophene (*28*) and 1-bromo-4-(triisopropylsilylethynyl)benzene (*29*) according to literature methods. We synthesized 1,2-bis(diphenylphosphino)ethane-bis(triphenylphosphine)nickel(0) as previously reported (*27*). Deuterated solvents were purchased from Cambridge Isotopes Laboratories Inc. Solvents used for air- and/or water-sensitive reactions (THF, toluene, pentane, and ether) were dried by passage through two columns of alumina and degassed by argon purge in a custom-built solvent purification system. ^1^H NMR spectra were obtained on a Varian NMR System 600-MHz spectrometer. Gel permeation chromatographs (GPCs) were obtained using a Waters 2690 Separation Module with two Agilent PLgel 5 μm MIXED-D columns running CHCl_3_/0.25% triethylamine as the eluent. MALDI spectra were obtained on a Bruker Microflex series MALDI-TOF using a matrix of dithranol-saturated chloroform.

### Synthesis of (dppe)Ni(triisopropylsilylethynylbenzene)bromide

In a nitrogen-filled glovebox, (dppe)Ni(PPh_3_)_2_ (0.200 g, 0.204 mmol) was dissolved in dry THF (0.95 ml). We slowly added 1-bromo-4-(triisopropylsilylethynyl)benzene (2.751 g, 8.149 mmol) via a syringe to the stirring solution. The reaction mixture was allowed to stir for 48 hours. Then, the solvent was evaporated off under reduced pressure. The resulting solid was fully precipitated with pentane (~25 ml), filtered, and dried under vacuum. For purification, the solid was dissolved in a minimum amount of toluene (~250 μl) and reprecipitated with pentane (~25 ml). The solid was recovered by filtration and dried under vacuum. The resulting orange solid (0.0994 g, 61%) was stored at −35°C. A summary of this synthesis and of the ^1^H NMR results for the resulting solid are shown in scheme S1.

### Polymerization of 2,5-dibromo-3-hexylthiophene

In a nitrogen-filled glovebox, 2,5-dibromo-3-hexylthiophene (0.473 g, 1.45 mmol) was dissolved in THF (3 ml). An isopropyl magnesium chloride–lithium chloride complex (1.08 ml, 1.3 M in THF) was then slowly added via syringe to the stirring solution. After 45 min, the reaction mixture was added to a stirring solution of **2** (0.046 g, 0.0.058 mmol, 10 ml of THF) in one shot. The polymerization was allowed to proceed for 3 min before being quenched by ethyl magnesium bromide (0.58 ml, 1 M in THF). The reaction mixture was stirred for 2 hours in the glovebox before being removed and quenched with methanol. The polymer was precipitated with ~50 ml of methanol and centrifuged. The supernatant was discarded, and the polymer was washed twice with methanol (~50 ml). The resulting polymer was dried under vacuum to give a black solid **P3HT-3S** (0.190 g, 79%) (GPC results, *M*_n_ = 7100, *M*_w_ = 9100, *Đ* = 1.3). A summary of this polymerization and of the ^1^H NMR results for **P3HT-3S** are shown in scheme S2.

### Removal of triisopropylsilyl-protecting group from terminal alkyne of P3HT-3

**P3HT-3S** (0.050 g, 0.0061 mmol) was dissolved in THF (1.0 ml) under an argon atmosphere. TBAF (0.07 ml, 1 M in THF) was added slowly via syringe. The reaction was allowed to proceed for about 15 hours. The polymer was precipitated in methanol (~50 ml) and centrifuged. The supernatant was discarded, and the polymer was washed twice with methanol (~50 ml). The resulting polymer was dried under vacuum to give a black solid **P3HT-4S** (0.041 g, 84%, quantitative deprotection) (GPC results, *M*_n_ = 6600, *M*_w_ = 8900, *Đ* = 1.3). A summary of this reaction and of ^1^H NMR results for **P3HT-4S** is shown in scheme S3.

### Click coupling of P3HT-4 and triethoxysilane-azide

Under an argon atmosphere, P3HT-**4** (0.019 mmol, 1 eq.), triethoxysilane-azide (0.093 mmol, 5 eq.), and copper iodide (0.056 mmol, 3 eq.) were dissolved in THF. DIPEA (0.56 mmol, 30 eq.) was added via syringe. The reaction mixture was heated to 40°C and allowed to stir for 2 days. The reaction mixture was cooled to room temperature. The product was precipitated out with ethyl acetate and centrifuged. The supernatant was discarded, and the product was washed with ethyl acetate. **TES-P3HT-S** was produced as a black solid with a yield of 81% (GPC results, *M*_n_ = 8800, *M*_w_ = 9900, *Đ* = 1.1). A summary of this reaction and of the ^1^H NMR results for **TES-P3HT-S** is shown in scheme S4.

### TES-P3HT with a longer chain length

A procedure analogous to the one described above was followed to produce **TES-P3HT-L**. **TES-P3HT-L** was obtained at a longer chain length than **TES-P3HT-S** by increasing the monomer-to-catalyst ratio during the initial polymerization step from 40:1 to 80:1. GPC results for the corresponding samples were as follows: **P3HT-3L**, *M*_n_ = 11,800, *M*_w_ = 16,500, *Đ* = 1.4; **P3HT-4L**, *M*_n_ = 11,200, *M*_w_ = 15,800, *Đ* = 1.4; and **TES-P3HT-L**, *M*_n_ = 10,600, *M*_*w*_ = 15,900, *Đ* = 1.5.

### MALDI spectra

MALDI-TOF mass spectrometry was used to identify polymer end groups (*31*). The MALDI mass distributions, measured in reflector mode, are shown below, where the correct repeat spacing of the peaks of 166 mass units is observed. On the basis of the spectrum in fig. S1, MALDI end group analysis confirms the presence of the TIPS-protected ethynylbenzene moiety on each polymer chain. However, there were two sets of peaks present in the mass distribution curve that could be reasonably attributed to two different functional groups on the chain end opposite the TIPS-ethynyl functionality. From the mass of the products, it was determined that these end groups were the desired ethyl group and also hydrogen. Hydrogen-terminated P3HT chains did not interfere with end group functionalization via click chemistry in subsequent transformations. MALDI end group analysis also confirmed the removal of the TIPS-group (fig. S2) and the successful incorporation of the triethoxysilane group (fig. S3).

MALDI spectra were obtained for the higher molecular weight P3HT analogs. At a higher molecular weight, polymers did not ionize easily. Therefore, spectra were measured in linear mode and did not provide as clean of spectra. End group analysis confirmed the desired functionalization at each of the three reaction steps (figs. S4 to S6).

### Substrate cleaning for single-molecule microscopy experiments

All reagents and solvents were purchased from Alfa Aesar, Fisher Scientific, and Sigma-Aldrich and used without further purification. No. 2 glass coverslips were cleaned in an ultrasound bath according to the following cycle: 15 min in Alconox detergent/5 min in Milli-Q water/15 min in acetone/5 min in Milli-Q water/15 min in a 1 M dilution of potassium hydroxide (KOH) in Milli-Q water/5 min in Milli-Q water/15 min in methanol/5 min in Milli-Q water/15 min in 1 M KOH dilution/5 min in Milli-Q water. After this cycle, the substrates were rinsed with Milli-Q water and then with methanol and finally dried with nitrogen. Then, they were plasma-etched for 3 min per side in a 0.2-mbar O_2_ atmosphere right before incubation.

### Single-end anchoring of single P3HT Chains

Single triethoxysilane-terminated P3HT chains were diluted in *o*-DCB to a ~2 nM concentration in THF. Then, hydrolysis of the triethoxysilane agents was promoted for 5 min in a THF/AcOH/H_2_0 (95:2.5:2.5 v/v) buffer, followed by reaction with the substrates for 1 hour in the same solution. Incubation was carried out in an ultrasound bath at 50°C to avoid aggregation and nonspecific binding of single chains. Posterior curing in vacuum for 1 hour was applied to evaporate residual water. Finally, the samples were rinsed twice with spectroscopy-grade *o*-DCB for 1 min in an ultrasound bath and dried with nitrogen. After incubation, the samples were either taken directly to the microscopy setup for imaging or stored in an oxygen-free glovebox for no longer than 4 days.

### Single-molecule data collection and analysis

Polymer-incubated glass substrates were assembled onto the brass chamber and flushed with nitrogen for 1 min and then filled with the corresponding degassed solvent (*o*-DCB or DMSO). A steady flux of degassed solvent from a reservoir vial was then maintained to avoid fast photodegradation of the samples. The flux was controlled by regulating the nitrogen pressure inside the reservoir vial. All solvents were degassed by bubbling nitrogen for at least 1 hour and kept tightly sealed until imaging. For the experiments involving the exchange from one solvent to another, the different solvents were injected into the reservoir vial subsequently when the previous solvent was running out. All samples were excited with a 473-nm laser in continuous wave operation (27 W/cm^2^) and imaged in epifluorescence mode. The excitation beam was circularly polarized to excite as many chromophores as possible. The beam was focused on the back-focal plane of an oil immersion objective (PlanApo, 60×, numerical aperture = 1.42, working distance = 0.17 mm). Any residual excitation light was filtered out with a dichroic beamsplitter (Semrock, 480-nm edge LaserMUX), and the collected PL emission was directed to an electron-multiplying charge-coupled device camera (Andor Ixon Ultra 897) cooled at −70°C. PL intensity movies of the samples were recorded with integration times of 100 ms.

### Single-molecule PL intensity trace analysis

For experiments in individual organic solvents, we collected 27 movies for sample TES-P3HT-S in DMSO (297 identified single chains) and 5 movies in *o*-DCB (514 single chains). For sample TES-P3HT-L, we collected 18 movies in DMSO (231 single chains) and 10 movies in *o*-DCB (764 single chains). The raw data were analyzed using the software iSMS (*60*) and custom-built Matlab algorithms to extract the single-chain intensity traces. Each intensity peak in the raw movies was fitted to a 2D Gaussian function. Next, the volume of the corresponding Gaussian was obtained for each peak, frame by frame, using a Matlab built-in function. The single-chain traces were then classified as in three main categories (classes I, II, and III), according to the following criteria:

**Class I:** (i) These traces followed a biexponential decay. (ii) They exhibited a single significant PL intensity peak within a certain characteristic time from the start of imaging, which was obtained from the average trace of each experiment. A peak was regarded as significant if its intensity was at least 1.2 kHz higher than any other local maximum. (iii) The highest intensity level was not a stable one; the criterion for regarding a level as stable was that at least a characteristic number of frames reached the peak intensity within a 0.6-kHz range. We estimated this value as twice the maximum amplitude of noise in the traces.

**Class II:** (i) There was at least one stable level. For a PL intensity level to be considered as stable, at least a characteristic number of frames had to reach this value within a 0.6-kHz range. (ii) The highest PL intensity level was higher than 1.2 kHz below the absolute maximum intensity of the trace.

**Class III:** (i) Any trace that did not meet any of the above conditions. The characteristic times and corresponding number of frames were calculated from the average traces of each experiment, as described in the algorithm below.

To categorize the intensity traces according to the previous classification, we analyzed each of them using the following algorithm in a home-built Matlab code:

1. An average PL intensity trace was obtained from the raw data of all the individual chains of each experiment. These average traces are shown in fig. S7. Each of them was fitted to a biexponential decay, from which the characteristic decay times, τ_1_ and τ_2_ were extracted. From these times, the average decay time (<τ>) was calculated as

$$<\tau >=(A_{1}\tau_{1}^{2}+A_{2}\tau_{2}^{2})/(A_{1}\tau_{1}+A_{2}\tau_{2}).$$

The corresponding parameters for each experiment are presented in table S1. Once the parameters were obtained, steps 2 to 11 were applied to each individual trace.

2. Noise was filtered from raw PL intensity traces using a Chung-Kennedy filter (*56*). Filtering parameters were a seven-frame window and a sensitivity factor *r* = 49.

3. Each individual trace was fitted to a biexponential function *f*(*t*) = *A*_1_exp(*k*_1_*t*)+*A*_2_exp(*k*_2_*t*).

4. A histogram of intensities was built for the corresponding trace. Each bin corresponded to an intensity range between 29 and 31 counts, which corresponds to 0.29 and 0.31 kHz, respectively, for an integration time of 100 ms.

5. Each peak in the histogram was associated to stable intensity levels if: (i) it was a local maximum and (ii) its frequency value corresponded to a number of frames equivalent to τ_c_ = τ_1_/2, where τ_1_ was the first characteristic time of the biexponential decay of the average trace of the corresponding experiment.

6. It was verified whether the fitted function (step 3) was a monotonically decreasing one.

7. It was verified whether local maxima within the trace occurred after a time equal to τ_c_, counting from the start of the measurement. In case there was any, it was also verified whether its intensity reached a value within a range of 120 counts (1.2 kHz) from the absolute maximum.

8. It was verified whether there were any stable levels within a 120-count range from the absolute maximum intensity of the trace.

9. Then, the trace was classified as class I if: (i) the fit was monotonically decreasing, (ii) there were no stable intensity levels around the absolute maximum intensity of the trace, (iii) both characteristic values, *k*_1_ and *k*_2_, of the fit were negative, (iv) the absolute maximum happened before τ_c_, and (v) there were no other peaks after τ_c_ and with an intensity within a range of 120 counts from the absolute maximum.

10. If any of the above conditions was not met, then the trace was classified as class II if: (i) it had at least one stable level and (ii) the peak intensity was part of a stable level.

11. If any of the conditions required for either of classes I and II, then the trace was classified as class III.

### Analysis of PL intensity traces from solvent-switching experiments

For experiments involving solvent exchange from DMSO to *o*-DCB, five movies were collected for sample TES-P3HT-S (312 single chains identified) and eight for sample TES-P3HT-L (335 single chains). An average trace was obtained for each movie and filtered using a 13-point median filter. Then, its first derivative was calculated with a four-point finite-difference method and filtered with the same filter. The moment of solvent-exchange was estimated as that at which the first derivative of the trace went above 100 Hz^2^ (one count per frame). All of the raw traces were synchronized at the same solvent-exchange frame and normalized. Then, a PL intensity histogram was built for every frame using the intensity values of all the traces. These intensity histograms were used for building the heat maps from Fig. 5 (A and B).

For experiments involving solvent exchange from *o*-DCB to DMSO and back to *o*-DCB, five movies were collected for sample TES-P3HT-S (482 single chains) and three movies for sample TES-P3HT-L (225 single chains). For these movies, the moment of DMSO injection was identified by the minimum in the second derivative of the average trace of each movie. This minimum had the condition of being around the shoulder caused by a sudden decrease of PL intensity at the moment of DMSO injection. The second injection (*o*-DCB) was identified as described above for the single-exchange experiments, but with a requirement of the first derivative being higher than 30 Hz^2^ rather than 100 Hz^2^ due to the partial photobleaching of the chain before the first injection. Both solvent-exchange frames were synchronized for all the traces, which required cutting some frames between them for some of the movies.

## Supplementary Material

http://advances.sciencemag.org/cgi/content/full/4/2/eaao5786/DC1

## SUPPLEMENTARY MATERIALS

Supplementary material for this article is available at http://advances.sciencemag.org/cgi/content/full/4/2/eaao5786/DC1 scheme S1. Synthesis of (dppe)Ni(triisopropylsilylethynylbenzene)bromide. scheme S2. Polymerization of 2,5-dibromo-3-hexylthiophene. scheme S3. Deprotection of P3HT-3. scheme S4. Click coupling of P3HT-4 and triethoxysilane-azide. fig. S1. MALDI spectrum of P3HT-3. fig. S2. MALDI spectrum of P3HT-4. fig. S3. MALDI spectrum of **TES-P3HT**. fig. S4. MALDI spectrum of P3HT-3H. fig. S5. MALDI spectrum of P3HT-4H. fig. S6. MALDI spectrum of **TES-P3HT-H**. fig. S7. Average PL intensity traces for each experiment in individual solvents and the corresponding normalized plots. fig. S8. Class I trace exhibiting a stepwise decay of the PL intensity. fig. S9. Average trace per each traces class for both molecular weights. fig. S10. Representative single-molecule PL intensity trace. fig. S11. Frequency maps for all of the traces from solvent-exchange experiments from *o*-DCB to DMSO. table S1. Characteristic decay times for the average PL intensity traces of each experiment. video S1. Single TES-P3HT-L molecule during solvent exchange from DMSO to *o*-DCB. video S2. Single TES-P3HT-S molecule during solvent exchange from DMSO to *o*-DCB.
