# Supplementary material for: Real-time observation of conformational switching in single conjugated polymer chains
Source: Sci Adv. 2018 Feb 16;4(2):eaao5786. doi: 10.1126/sciadv.aao5786 (PMC5817931; doi:10.1126/sciadv.aao5786)
Supplement: http://advances.sciencemag.org/cgi/content/full/4/2/eaao5786/DC1 [file supp_4_2_eaao5786__index.html]

Science Advances | Science Advances

## Supplementary Materials

**This PDF file includes:**

- scheme S1. Synthesis of (dppe)Ni(triisopropylsilylethynylbenzene)bromide.
- scheme S2. Polymerization of 2,5-dibromo-3-hexylthiophene.
- scheme S3. Deprotection of P3HT-3.
- scheme S4. Click coupling of P3HT-4 and triethoxysilane-azide.
- fig. S1. MALDI spectrum of P3HT-3.
- fig. S2. MALDI spectrum of P3HT-4.
- fig. S3. MALDI spectrum of **TES-P3HT**.
- fig. S4. MALDI spectrum of P3HT-3H.
- fig. S5. MALDI spectrum of P3HT-4H.
- fig. S6. MALDI spectrum of **TES-P3HT**-H.
- fig. S7. Average PL intensity traces for each experiment in individual solvents and the corresponding normalized plots.
- fig. S8. Class I trace exhibiting a stepwise decay of the PL intensity.
- fig. S9. Average trace per each traces class for both molecular weights.
- fig. S10. Representative single-molecule PL intensity trace.
- fig. S11. Frequency maps for all of the traces from solvent-exchange experiments from *o*-DCB to DMSO.
- table S1. Characteristic decay times for the average PL intensity traces of each experiment.
- Legends for videos S1 and S2

Download PDF

**Other Supplementary Material for this manuscript includes the following:**

- video S1a and video S1b (.avi format). **Single TES-P3HT-L molecule during solvent exchange from DMSO to *o*-DCB.** A sudden increase in the PL intensity is observed at the moment of solvent exchange, at ~ 4.6 seconds from the start of the video. This video corresponds to the PL intensity trace shown in fig. S10. Video S1a is played at normal speed and video S1b is played at 10 times the speed of the measurement in order to minimize its length.
- video S2a and video S2b (.avi format). **Single TES-P3HT-S molecule during solvent exchange from DMSO to *o*-DCB.** A sudden increase in the PL intensity is observed at the moment of solvent exchange, at ~3.1 seconds from the start of the video. This video corresponds to the PL intensity trace shown in Fig. 4A. Video S2a is played at normal speed and video S2b is played at 10 times the speed of the measurement in order to minimize its length.

**Files in this Data Supplement:**

- Adobe PDF - aao5786\_SM.pdf
