# Supplementary material for: Real-time observation of conformational switching in single conjugated polymer chains
Source: Sci Adv. 2018 Feb 16;4(2):eaao5786. doi: 10.1126/sciadv.aao5786 (PMC5817931; doi:10.1126/sciadv.aao5786)
Supplement: http://advances.sciencemag.org/cgi/content/full/4/2/eaao5786/DC1 [file aao5786_SM.pdf]

Science Advances

AAAS

advances.sciencemag.org/cgi/content/full/4/2/eaao5786/DC1

Supplementary Materials for

Real-time observation of conformational switching in single conjugated polymer chains

Francisco Tenopala-Carmona, Stephanie Fronk, Guillermo C. Bazan, Ifor D. W. Samuel, J. Carlos Penedo

Published 16 February 2018, *Sci. Adv.* **4**, eaao5786 (2018)
DOI: 10.1126/sciadv.aao5786

The PDF file includes:

- scheme S1. Synthesis of (dppe)Ni(triisopropylsilylethynylbenzene)bromide.
- scheme S2. Polymerization of 2,5-dibromo-3-hexylthiophene.
- scheme S3. Deprotection of P3HT-3.
- scheme S4. Click coupling of P3HT-4 and triethoxysilane-azide.
- fig. S1. MALDI spectrum of P3HT-3.
- fig. S2. MALDI spectrum of P3HT-4.
- fig. S3. MALDI spectrum of **TES-P3HT**.
- fig. S4. MALDI spectrum of P3HT-3H.
- fig. S5. MALDI spectrum of P3HT-4H.
- fig. S6. MALDI spectrum of **TES-P3HT**-H.
- fig. S7. Average PL intensity traces for each experiment in individual solvents and the corresponding normalized plots.
- fig. S8. Class I trace exhibiting a stepwise decay of the PL intensity.
- fig. S9. Average trace per each traces class for both molecular weights.
- fig. S10. Representative single-molecule PL intensity trace.
- fig. S11. Frequency maps for all of the traces from solvent-exchange experiments from *o*-DCB to DMSO.
- table S1. Characteristic decay times for the average PL intensity traces of each experiment.
- Legends for videos S1 and S2

Other Supplementary Material for this manuscript includes the following:
(available at advances.sciencemag.org/cgi/content/full/4/2/eaao5786/DC1)

- video S1 (.avi format). Single TES-P3HT-L molecule during solvent exchange from DMSO to *o*-DCB.
- video S2 (.avi format). Single TES-P3HT-S molecule during solvent exchange from DMSO to *o*-DCB.

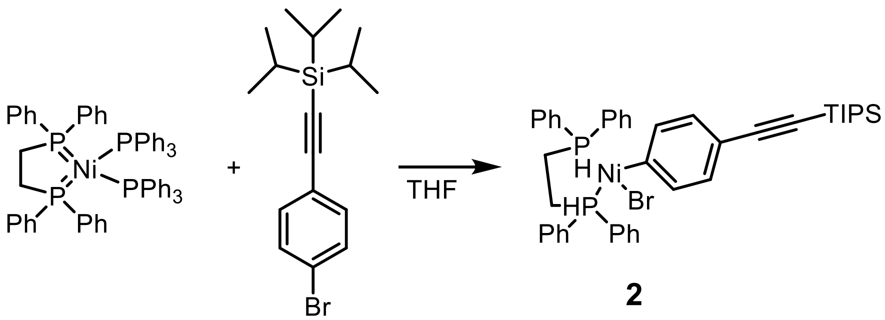

scheme S1. Synthesis of (dppe)Ni(triisopropylsilylethynylbenzene)bromide. ^{1}HNMR results for solid **2** (600 MHz, C_6D_6) δ (ppm): 8.00 (t, 3H), 7.40 (t, 2H), 7.25 (dd, 4H), 7.10 (m, 6H), 7.00 (m, 1H), 6.95 (t, 2H), 6.85 (t, 3H), 1.70 (ddd, 2H), 1.20 (d, 14H), 1.15 (m, 3H).

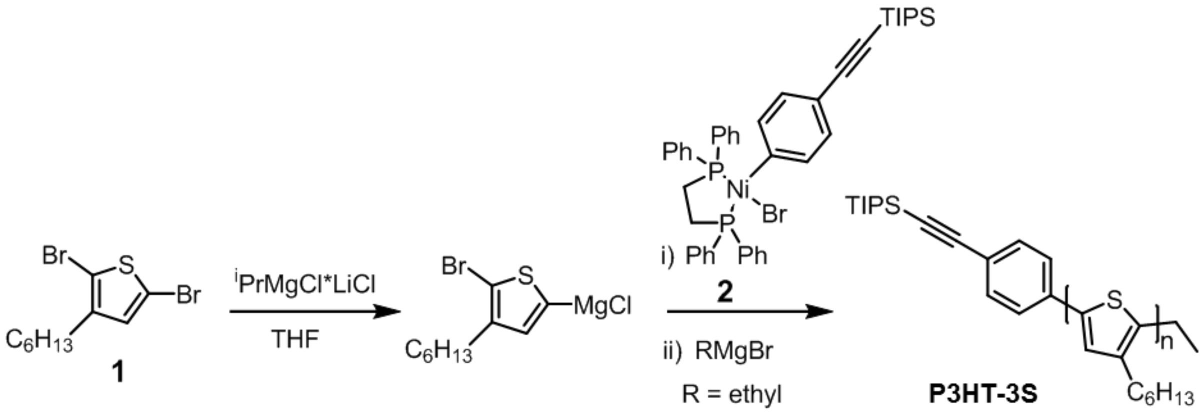

scheme S2. Polymerization of 2,5-dibromo-3-hexylthiophene. ^{1}HNMR results for **P3HT-3S** (600MHz, $CDCl_3$) δ (ppm): 7.5 (d, 0.04H), 7.45 (d, 0.04H), 6.95 (s, 1H), 2.8 (t, 2H), 1.7 (t, 2H), 1.4 (t, 2H), 1.3 (t, 4H), 1.15 (s, 0.44H), 0.9 (t, 3H). ^{13}CNMR (600 MHz, $CDCl_3$) δ (ppm): 140.5, 134.5, 131.0, 128.5, 31.5, 30.5, 29.5, 29.3, 22.5, 14.0.

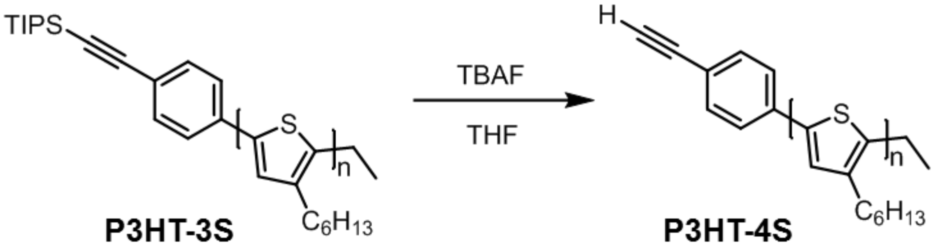

scheme S3. Deprotection of P3HT-3. ^{1}HNMR results for **P3HT-4S** (600MHz, $CDCl_3$) δ (ppm): 7.5 (d, 0.04H), 7.45 (d, 0.04H), 6.95 (s, 1H), 3.1 (s, 0.02H), 2.8 (t, 2H), 1.7 (t, 2H), 1.4 (t, 2H), 1.3 (t, 4H), 0.9 (t, 3H). ^{13}CNMR (600 MHz, $CDCl_3$) δ (ppm): 140.5, 134.5, 131.0, 128.5, 31.5, 30.5, 29.5, 29.3, 22.5, 14.0.

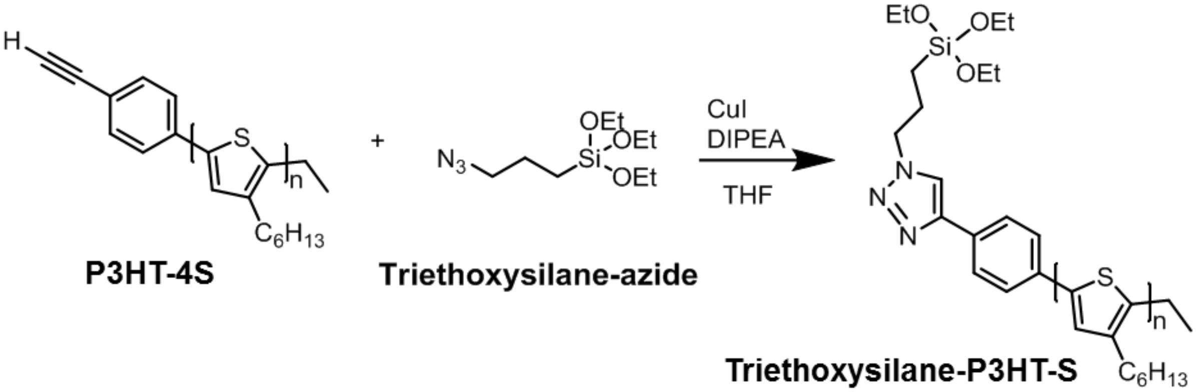

scheme S4. Click coupling of P3HT-4 and triethoxysilane-azide. ^{1}HNMR results for **TES-P3HT-S** (600MHz, $CDCl_3$) δ (ppm): 7.83 (d, 0.03H), 7.79 (s, 0.01H), 7.64 (d, 0.03H), 6.97 (s, 1H), 4.41 (t, 0.02), 3.82 (m, 0.07H), 2.8 (t, 2H), 1.7 (t, 2H), 1.4 (t, 2H), 1.3 (t, 4H), 0.9 (t, 3H), 0.64 (t, 0.05H).

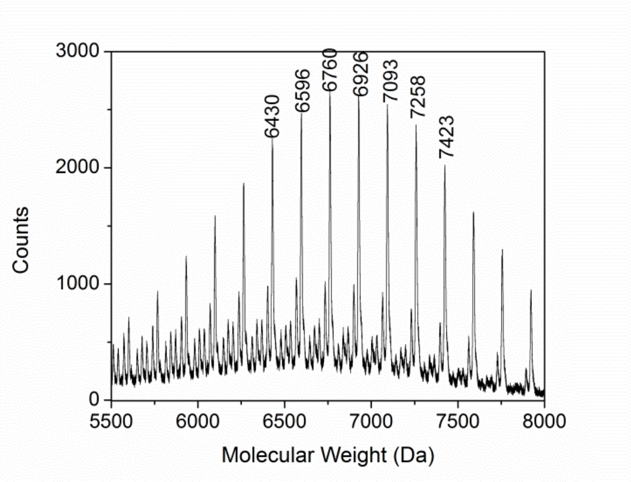

fig. S1. MALDI spectrum of P3HT-3.

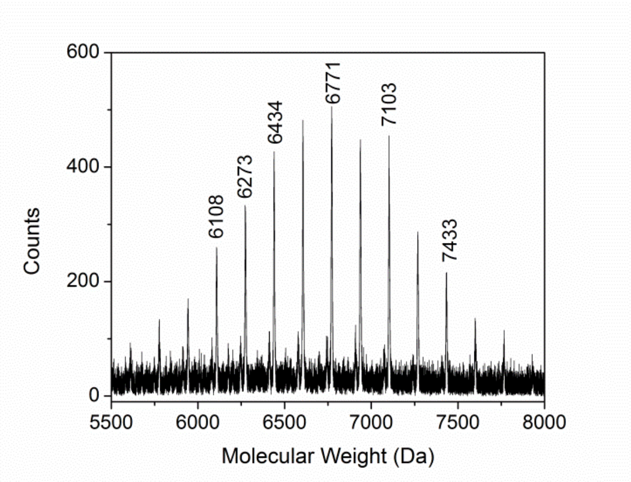

fig. S2. MALDI spectrum of P3HT-4.

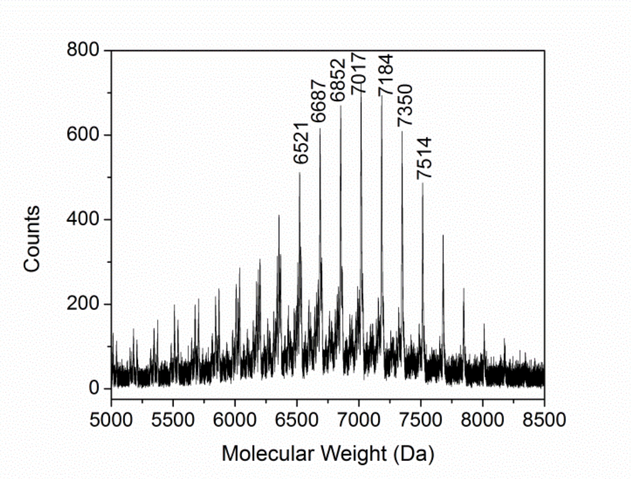

fig. S3. MALDI spectrum of TES-P3HT.

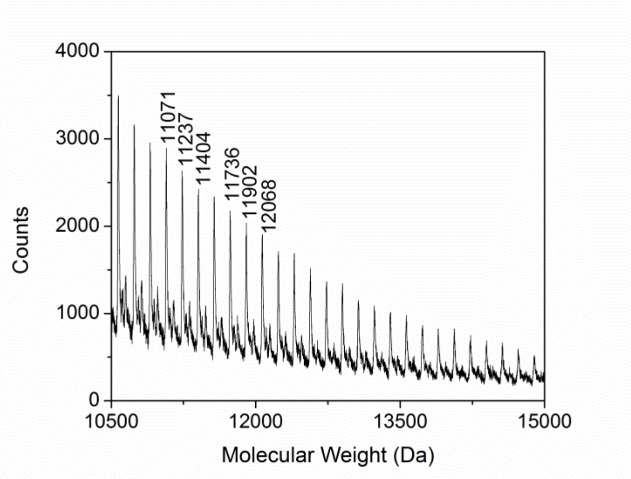

fig. S4. MALDI spectrum of P3HT-3H.

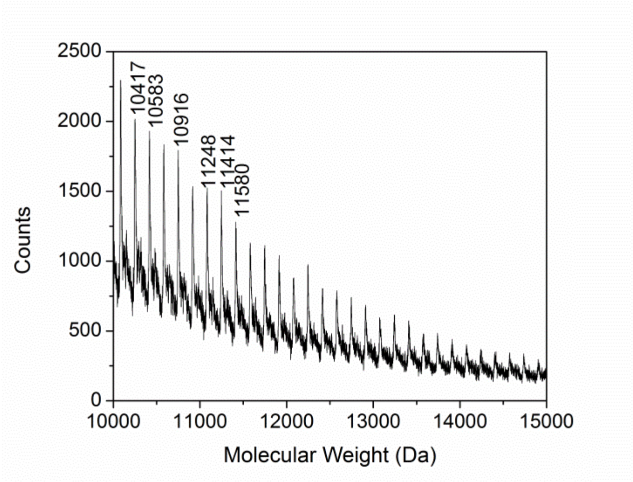

fig. S5. MALDI spectrum of P3HT-4H.

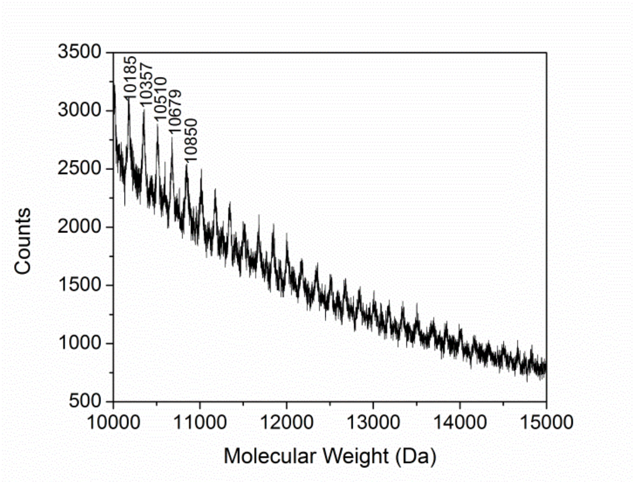

fig. S6. MALDI spectrum of TES-P3HT-H.

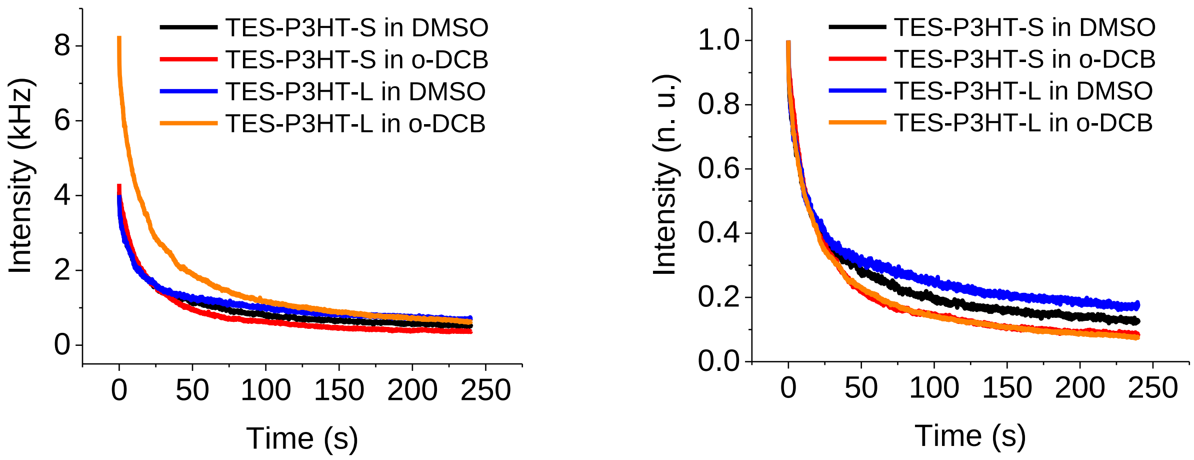

fig. S7. Average PL intensity traces for each experiment in individual solvents and the corresponding normalized plots. It can be seen on the left panel that the initial PL intensity for the sample TES-P3HT-L in o-dichlorobenzene is about two-fold higher than that for the same sample in DMSO, as well as than the sample TES-P3HT-S in both solvents. It can be seen from the right panel that despite the difference in the magnitude of the PL intensity, the photobleaching rates are very similar for both samples in o-dichlorobenzene. It can also be seen that both samples have lower photobleaching rates in DMSO, being it lower for sample TES-P3HT-L.

table S1. Characteristic decay times for the average PL intensity traces of each experiment. Uncertainties in τ_1, τ_2, A_1, and A_2 represent the error in the fit and were propagated according to equation $\langle\tau\rangle = (A_1\tau_1^2+A_2\tau_2^2)/(A_1\tau_1+A_2\tau_2)$ for τ.

**Experiment**	**<τ> (s)**	**τ_1 (s)**	**A_1**	**τ_2 (s)**	**A_2**
8.8 kDa o-DCB	46.6 ± 0.5	11.5 ± 0.1	0.620 ± 0.003	57.9 ± 0.6	0.380 ± 0.003
8.8 kDa DMSO	55.8 ± 0.5	8.6 ± 0.1	0.537 ± 0.002	63.2 ± 0.5	0.463 ± 0.002
10.6 kDa o-DCB	51.2 ± 0.4	10.7 ± 0.1	0.569 ± 0.002	60.7 ± 0.5	0.431 ± 0.002
10.6 kDa DMSO	78.4 ± 1.0	8.9 ± 0.1	0.617 ± 0.002	89.6 ± 1.2	0.383 ± 0.002

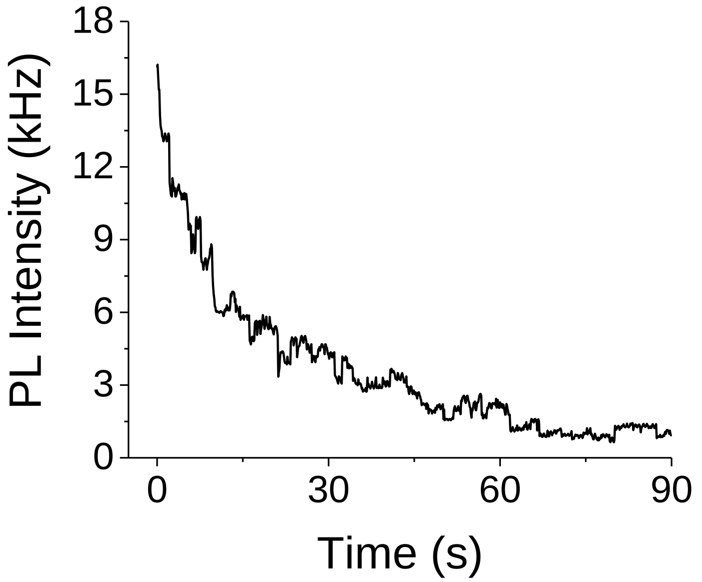

fig. S8. Class I trace exhibiting a stepwise decay of the PL intensity.

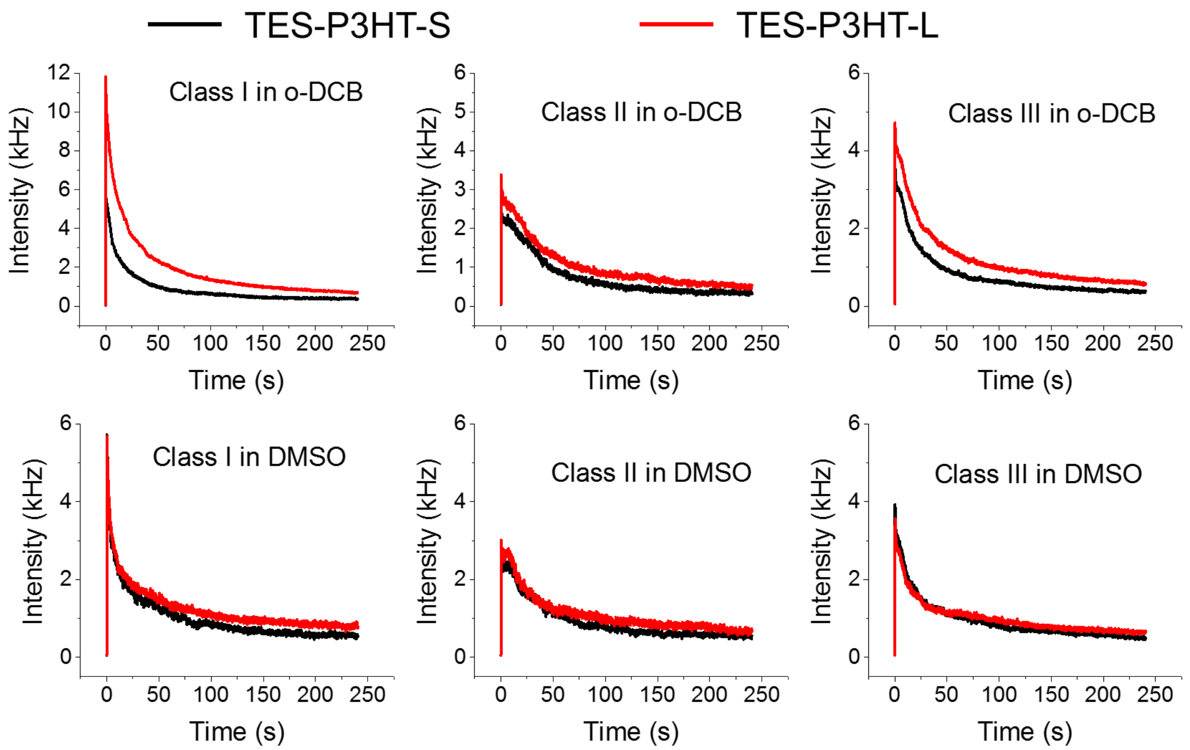

fig. S9. Average trace per each traces class for both molecular weights. The difference in the initial PL intensity between samples TES-P3HT-S and TES-P3HT-L is higher for Class I in o-dichlorobenzene than that of the other classes in the same solvent. It can also be seen that the same difference is much lower for the corresponding experiments in DMSO.

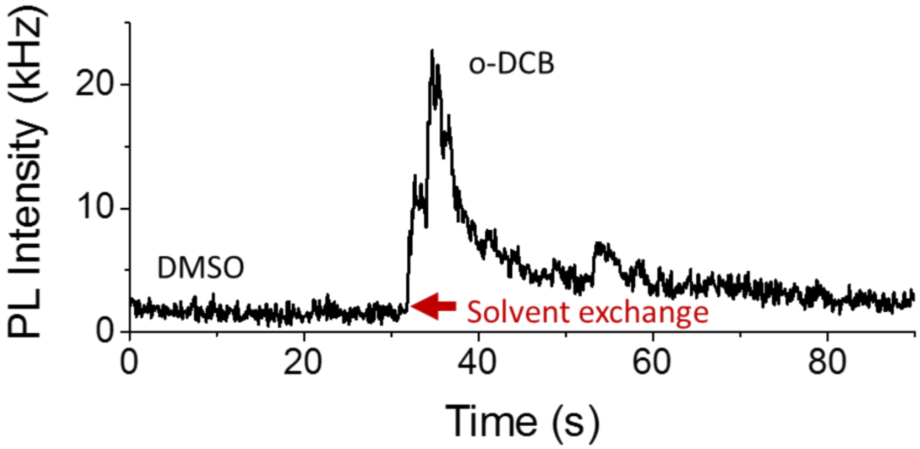

fig. S10. Representative single-molecule PL intensity trace. The abrupt change in fluorescent emission takes place in the same TES-P3HT-L chain upon switching at ~32 seconds from a poor solvent environment (DMSO) to a good solvent (o-DCB). A >6-fold change in fluorescent emission is exhibited, reaching the peak PL intensity in ~5 seconds. Such increase is followed by continuous monotonic decrease in signal due to chromophore photobleaching. In contrast to the trace from polymer TES-P3HT-S shown in Fig. 4A, this PL intensity trace takes a longer time in reaching the peak, even exhibiting an intermediate intensity level.

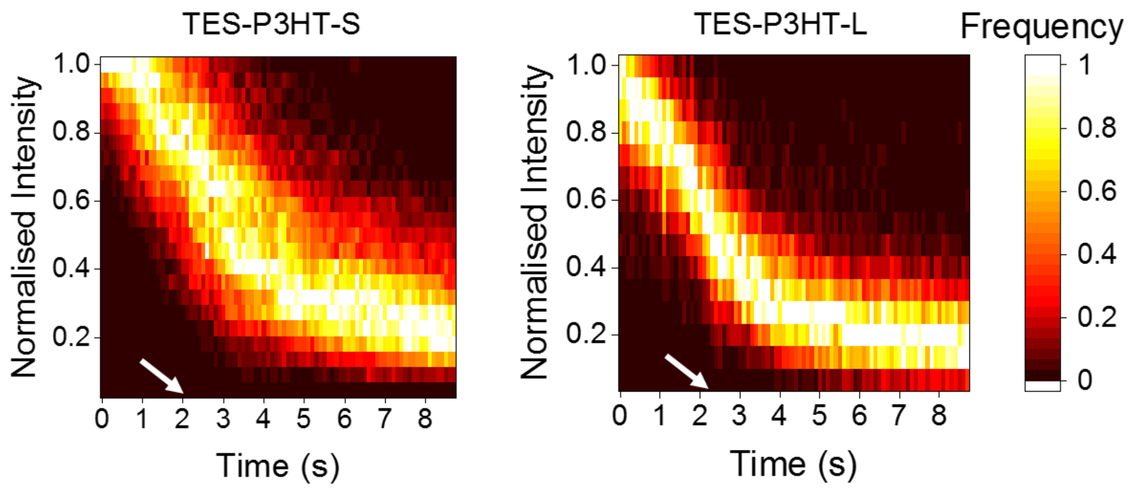

fig. S11. Frequency maps for all of the traces from solvent-exchange experiments from *o*-DCB to DMSO. The white arrows indicate the time at which DMSO was injected. An inflection point can be observed in the trend of the traces. This change in curvature is due to the sudden decrease in the PL intensity caused by DMSO injection.

video S1. Single TES-P3HT-L molecule during solvent exchange from DMSO to *o*-DCB. A sudden increase in the PL intensity is observed at the moment of solvent exchange, at ~ 4.6 seconds from the start of the video. This video corresponds to the PL intensity trace shown in fig. S10. Video S1a is played at normal speed and video S1b is played at 10 times the speed of the measurement in order to minimize its length.

video S2. Single TES-P3HT-S molecule during solvent exchange from DMSO to *o*-DCB. A sudden increase in the PL intensity is observed at the moment of solvent exchange, at ~3.1 seconds from the start of the video. This video corresponds to the PL intensity trace shown in

Fig. 4A. Video S2a is played at normal speed and video S2b is played at 10 times the speed of the measurement in order to minimize its length.
